# Friction factor for turbulent open channel flow covered by vegetation

**DOI:** 10.1038/s41598-019-41477-7

**Published:** 2019-03-26

**Authors:** Wei-Jie Wang, Wen-Qi Peng, Wen-Xin Huai, Gabriel G. Katul, Xiao-Bo Liu, Xiao-Dong Qu, Fei Dong

**Affiliations:** 1 State Key Laboratory of Simulation and Regulation of Water Cycle in River Basin, China Institute of Water Resources and Hydropower Research, Beijing, 100038 China; 2 Department of Water Environment, China Institute of Water Resources and Hydropower Research, Beijing, 100038 China; 3 State Key Laboratory of Water Resources and Hydropower Engineering Science, Wuhan University, Wuhan, Hubei 430072 China; 4 Nicholas School of the Environment, Duke University, Durham, North Carolina 27708 USA; 5 Department of Civil and Environmental Engineering, Duke University, Durham, North Carolina 27708 USA

## Abstract

The need for operational models describing the friction factor *f* in streams remains undisputed given its utility across a plethora of hydrological and hydraulic applications concerned with shallow inertial flows. For small-scale roughness elements uniformly covering the wetted parameter of a wide channel, the Darcy-Weisbach *f* = 8(u_*_/*U*_*b*_)^2^ is widely used at very high Reynolds numbers, where u_*_ is friction velocity related to the surface kinematic stress, *U*_*b*_ = *Q*/*A* is bulk velocity, *Q* is flow rate, and *A* is cross-sectional area orthogonal to the flow direction. In natural streams, the presence of vegetation introduces additional complications to quantifying *f*, the subject of the present work. Turbulent flow through vegetation are characterized by a number of coherent vortical structures: (i) von Karman vortex streets in the lower layers of vegetated canopies, (ii) Kelvin-Helmholtz as well as attached eddies near the vegetation top, and (iii) attached eddies well above the vegetated layer. These vortical structures govern the canonical mixing lengths for momentum transfer and their influence on *f* is to be derived. The main novelty is that the friction factor of vegetated flow can be expressed as *f*_*v*_ = 4*C*_*d*_(*U*_*v*_/*U*_*b*_)^2^ where *U*_*v*_ is the spatially averaged velocity within the canopy volume, and *C*_*d*_ is a local drag coefficient per unit frontal area derived to include the aforemontioned layer-wise effects of vortical structures within and above the canopy along with key vegetation properties. The proposed expression is compared with a number of empirical relations derived for vegetation under emergent and submerged conditions as well as numerous data sets covering a wide range of canopy morphology, densities, and rigidity. It is envisaged that the proposed formulation be imminently employed in eco-hydraulics where the interaction between flow and vegetation is being sought.

## Introduction

Since its inception by Darcy (in 1857) and Weisbach (in 1845), the now called Darcy-Weisbach equation for determining frictional losses in open channels (and pipes) is considered ‘standard’ provided its associated friction factor coefficient $f=8{({u}_{\ast }/{U}_{b})}^{2}$ is known, where ${u}_{\ast }=\sqrt{g{R}_{h}{S}_{f}}$ is the friction velocity related to the kinematic ‘bed stress’, *g* is the gravitational acceleration, *R*_*h*_ is the hydraulic radius, *S*_*f*_ is the frictional slope or energy grade-line that converges to the bed-slope *S*_*o*_ for uniform flow, ${U}_{b}=Q/A$ is the bulk or time and area-averaged velocity determined from the flow rate *Q* and cross-sectional area $A=B{h}_{w}$ (assuming rectangular section) orthogonal to the flow direction, *B* is the channel width and *h*_*w*_ is the water depth approximating *R*_*h*_ when $B\gg {h}_{w}$. The definition of *f* can be combined with the estimate of u_*_ to yield

1 $${S}_{f}=\frac{f{U}_{b}^{2}}{8g{R}_{h}}.$$

In classical hydraulics, the work of Moody, Nikuradse and many others established *f* to vary with two dimensionless quantities: the relative roughness *r*/*R*_*h*_ and a bulk Reynolds number ${R}{{e}}_{b,h}={U}_{b}{R}_{h}/\nu$, where *ν* is the kinematic viscosity of water. This expression is generally accepted in pipe- and open channel- flows above small-scale roughness elements where $r/{h}_{w}\ll 1$. At very high *Re*_*b*,*h*_, *f* becomes independent of *Re*_*b*,*h*_ and is presumed to abide by the so-called Strickler scaling $f\sim {(r/{R}_{h})}^{1/3}$^1–3^. An immediate consequence of this result is that when *r* is a priori known, *f* and subsequently *S*_*f*_ can be determined from Eq. 1.

Operationally, such an expression for *S*_*f*_ can be used to mathematically close the combined continuity and unsteady shallow water flow equations (i.e., the Saint-Venant) to predict *h*_*w*_ and *U*_*b*_ in a plethora of hydrological and hydraulics applications. Example applications include overland flow from bare to vegetated patches, dam-break and similar shallow inertial flows, flash-flood runoff in ephemeral streams, tsunamis on coastal plains, to name a few^4–11^. It is now recognized that such naive view of *f* cannot be juxtaposition to streams covered by large roughness values ($r/{h}_{w} > 0.1$) such as shallow flow over gravel beds^12–17^ or vegetation^18–20^ and frames the scope of the work here. In fact, a number of authors are calling for the abandonment of such an approach altogether in such situations^21,22^.

The main theoretical novelty is to arrive at a new expression for *f* whose generic form resembles the conventional $f=8{({u}_{\ast }/{U}_{b})}^{2}$ for steady-uniform flow through or above dense canopies. Specifically, it is shown that under certain simplifying assumptions, the canopy-related *f* (hereafter referred to as *f*_*v*_) is given by

2 $${f}_{v}=4{C}_{d}{(\frac{{U}_{v}}{{U}_{b}})}^{2},$$

where *U*_*v*_ is the spatially averaged velocity within the canopy volume, and *C*_*d*_ is a local drag coefficient per unit frontal area derived to include the layer-wise effects of vortical structures within and above the canopy along with key vegetation properties. For this expression to be used in practice, an estimate of *U*_*v*_/*U*_*b*_ and *C*_*d*_ are required. The derivation of Eq. 2 and estimates of *C*_*d*_ and *U*_*v*_/*U*_*b*_ are first presented followed by a comparison between model predictions and measurements of *f*_*v*_. Comparisons with other widely used models of *f*_*v*_ are also featured demonstrating that the proposed formulation appears superior to all prior formulations across a wide-range of experiments and canopy-flow configurations.

### Background

To progress on the description of *f*_*v*_ for canopy flows, a number of studies have been conducted to explore connections between the shape of the mean velocity profile *u*(*z*) and its depth-integrated value defined as

3 $${U}_{b}\approx \frac{1}{{h}_{w}}\,{\int }_{0}^{{h}_{w}}\,u(z)dz,$$

in wide rectangular channels, where *z* is the distance from the channel bottom^23–30^. These studies naturally bridge the dominant vortical structures^31,32^ to *u*(*z*)^12,33–35^ and subsequently to *U*_*b*_ and *f*_*v*_. Other studies have focused on the force balance and stresses acting on the vegetation elements as well as *R*_*h*_ to arrive at u_*_ and *f*_*v*_^36–38^. Various resistance models for flow within and above vegetated elements were proposed that used bulk flow measurements or combination of models and measurements to determine *f* ^9,39–56^. The emerging picture from all these studies is that key vortical structures impact the shape of *u*(*z*) across various canopy layers and in the vegetation free layer for submerged vegetation. The relation between these vortical structures and *u*(*z*) is now reviewed.

### Review of the mean velocity profile within and above canopies

**Figure 1:**
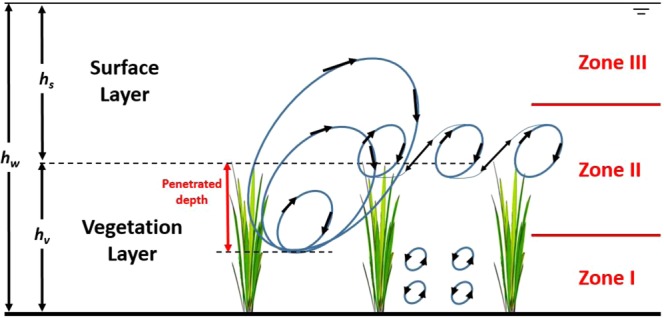
The key vortical structures in different regions of a vegetated zone in a wide channel where the vegetation is submerged. The vegetation height *h*_*v*_ and the water depth *h*_*w*_ are also presented. The surface (i.e. vegetation free zone) and vegetation layers are defined here.

For stationary and planar-homogeneous flow in a wide channel covered by a densely vegetated canopy, the flow region *h*_*w*_ can be decomposed into three zones according to the dominant sizes of the vortices in each zone as shown in Fig. 1.

Zone I forms near the channel bottom where von Karman vortex streets dominate the energetics of turbulence in the vertical direction. The mean velocity in zone I can be approximated by a constant given as^41,57,58^

4 $${u}_{I}=\sqrt{\frac{2g{S}_{f}}{{C}_{d}mD}},$$

where *C*_*d*_ is a local drag coefficient as before, *m* is the number of rods or vegetation stems per unit ground area, and *D* is the frontal width of vegetation elements.

Zone II spans the vegetation top and is dominated by attached eddies to a zero-plane displacement as well as mixing-layer eddies (though those types of eddies do not co-exist in space but both impact time-averaged statistics). When explained by the mixing layer analogy, the mean velocity is given by

5 $$\frac{{u}_{II}(z)}{{u}_{v,top}}=1+\,\tanh \,(\frac{z-{h}_{v}}{{L}_{mix}}),$$

where *u*_*v*,*top*_ is the mean velocity at the top of the vegetation elements, *h*_*v*_ is the vegetation height, *L*_*mix*_ is a characteristic energetic eddy size generated from Kelvin-Helmholtz instabilities^34^.

Zone III resembles a canonical turbulent boundary layer above a rough ground surface and is commonly represented by

6 $${u}_{III}(z)=\frac{{u}_{\ast ,sur}}{\kappa }\,\mathrm{ln}\,(\frac{z-d}{{z}_{0}}),$$

where the friction velocity ${u}_{\ast ,sur}\approx \sqrt{g{h}_{s}{S}_{f}}$ for the surface layer (as before) whose thickness is *h*_*s*_, *z*_*o*_ is the momentum roughness length (linked to *r* or *h*_*v*_), *d* is the zero-plane displacement height and is related to the penetration depth, *κ* is the von Karman constant, and ${h}_{s}={h}_{w}-{h}_{v}$ when ${h}_{w}/{h}_{v} > 1$ (submerged vegetation case).

Due to the presence of vegetation, the addition of zones I and II introduce major distortions to the expected *u*(*z*) shape when compared to a typical rough boundary (i.e. zone III) where *f*_*v*_ primarily varies with *r*/*h*_*w*_ and *Re*_*b*,*h*_ as before. When $r/{h}_{w}\ll 1$ (in zone III), *r* may be linked to *z*_0_ using the scaling relation $r\sim {z}_{o}^{1/6}$ discussed elsewhere^12^. The aforementioned distortions to *u*(*z*) and the possibility of deriving a general expression for *f*_*v*_ to be used in operational models for emergent and submerged vegetation is the main goal of the work here.

## Derivation of a Friction Factor for Vegetated Flow

The goal of the derivation here is to arrive at a new formula for *f*_*v*_ whose generic form resembles the conventional $f=8{({u}_{\ast }/{U}_{b})}^{2}$ and is given by

7 $${f}_{v}=8{(\frac{{u}_{\ast ,v}}{{U}_{b}})}^{2}=\frac{8g{S}_{v}{R}_{v}}{{U}_{b}^{2}}.$$

This definition necessitates estimates of the friction velocity for the vegetated flow ${u}_{\ast v}=\sqrt{g{S}_{v}{R}_{v}}$, where *S*_*v*_ denotes the energy grade-line slope caused by the presence of vegetation, *R*_*v*_ denotes a new vegetation-related hydraulic radius, and *U*_*b*_ is, as before, the bulk flow velocity averaged over the entire cross-sectional area but adjusted for the finite porosity of the vegetation medium.

Section 2.1 derives the vegetation-related energy slope *S*_*v*_ and Section 2.2 derives the vegetation-related hydraulic radius *R*_*v*_. The outcome is then compared to a wide range of published data sets and prior formulations derived from fitting to other published data sets.

### Derivation of vegetation-related energy slope

Throughout, it is assumed that the flow is steady and uniform occurring within a wide rectangular open channel characterized by width *B* and bed slope *S*_*o*_. The case considered here is when vegetation is sufficiently dense so that the overall friction factor is mainly due to *f*_*v*_ not bed and side-wall friction and the frictional slope is *S*_*v*_. The vegetation is further assumed to be cylindrical with height *h*_*v*_, diameter *D*, and density *m* defined by the number of cylinders per unit area as before. The vegetation can be in one of two-states: submerged ($\alpha  < 1$) or emergent ($\alpha  > 1$) as characterized by the degree of submergence $\alpha ={h}_{v}/{h}_{w}$. For a control volume above a unit bed area extending from the channel bed to the water surface, the mean momentum balance in the streamwise direction reduces to a force-balance between (i) the gravitational contribution of the water weight along the streamwise direction for a unit ground area (=*F*_*w*_), and (ii) the drag force per unit ground area (=*F*_*v*_) caused by the presence of vegetation stems resisting the flow. The *F*_*w*_ is given by

8 $${F}_{w}=\rho g{S}_{v}{V}_{w},$$

where *ρ* is the water density, *V*_*w*_ is the volume of flowing water per unit ground area determined from the entire domain volume reduced by the volume occupied by the vegetation elements. The ${V}_{w}={h}_{w}\,(1-\alpha \varphi )$ for submerged conditions (i.e. $\alpha  < 1$) and ${V}_{w}={h}_{w}\,(1-\varphi )$ for emergent vegetation (i.e. $\alpha  > 1$), where $\varphi$ is the volume fraction of the vegetation. When ignoring wall stresses relative to the drag force imposed by the dense vegetation and assuming a quadratic-law for *F*_*v*_ yields

9 $${F}_{v}={C}_{d}{A}_{v}\frac{\rho {U}_{ref}^{2}}{2},$$

where *A*_*v*_ is frontal area of vegetation given by ${A}_{v}=mD{h}_{v}$ for $\alpha  < 1$ and ${A}_{v}=mD{h}_{w}$ for $\alpha  > 1$, *C*_*d*_ in Eq. (9) is a ‘*local spatially*-*averaged*’ non-constant drag coefficient that must account for the effects of the vegetation on the flow as discussed elsewhere^55^, and *U*_*ref*_ is a reference velocity representing the flow within the vegetation section sensing the drag elements. A number of plausible velocities are now introduced to represent *U*_*ref*_: (a) The bulk velocity in the vegetation layer (VL, zones I + II) sensing the drag effects that can be estimated from ${U}_{v,b}={Q}_{v}/(B{h}_{v})$^59–61^. (b) The pore velocity in VL calculated based on a spatially-averaged value ${U}_{v,p}={Q}_{v}/({B}_{p}{h}_{v})$, where the effective width used for the pore velocity is ${B}_{p}=B(1-\varphi )$^36,51,62–65^. (c) The constricted velocity in a classical staggered array ${U}_{v,c}={Q}_{v}/({B}_{c}{h}_{v})$ with a characteristic constricted width ${B}_{c}=B(1-D/{L}_{s,stag})$^55^, where *L*_*s*,*stag*_ is the spacing distance defined in Etminan *et al*.’ work^55^. (d) The separation velocity ${U}_{v,s}={k}_{p}{U}_{v,p}$ where *k*_*p*_ is a kinetic energy of a subgrid scale (SGS) for a Smagorinsky model^55^. Due to different vegetation arrangements used in the experiments analyzed here (Section 3 and 4), the spatially-averaged pore velocity was adopted (i.e. ${U}_{ref}={U}_{v,p}$). Detailed analysis shows that this choice of *U*_*ref*_ along with a vegetation-related hydraulic radius resulting in a new Reynolds number perform reasonably when compared to many experiments (discussed later). Hence, from the force balance ${F}_{w}={F}_{v}$, the vegetation-related friction slope is given by

10 $${S}_{v}=\frac{{C}_{d}{A}_{v}}{2g{V}_{w}}{U}_{v,p}^{2},$$

where *C*_*d*_ and *U*_*v*,*p*_ remain to be determined.

### Derivation of a vegetation-related hydraulic radius *R*_*v*_

Many studies treat flow resistance of a vegetated canopy as equivalent to a ‘bed stress’ assuming energy losses occur due to a ‘vegetated bed’. This equivalence is convenient as it leads to a hydraulic radius related to flow depth *h*_*w*_ or surface layer depth *h*_*s*_^9,41,44,66^ without considering the details of the vegetation configuration such as frontal area associated with $\varphi$, *m* and *D*. However, the flow resistance by vegetation is mainly dominated by form drag and is directly linked to the frontal area of the rods. The hydraulic radius may be revised to account for these vegetation-related features. Here, a vegetation-related hydraulic radius *R*_*v*_ is proposed by extending the work of Cheng^36^ from emergent condition to submerged cases, where the vegetation-related hydraulic radius *R*_*v*_ is now defined by the ratio of the whole flow volume *V*_*w*_ per unit ground area to the vegetation-fluid contact *A*_*resistance*_ per unit ground area and can be expressed as

11 $${R}_{v}=\frac{{V}_{w}}{{A}_{{resistance}}}.$$

From Eq. (9), the vegetation resistance is caused by its form drag, which is a function of the frontal vegetated area *A*_*v*_ per unit ground area. Hence, the vegetation-related hydraulic radius is

12 $${R}_{v}=\frac{{V}_{w}}{{A}_{v}}.$$

Since both *V*_*w*_ and *A*_*v*_ are defined per unit ground area, the ground area cancels in the definition of *R*_*v*_.

### Derivation of the friction formula for flow through submerged and emergent vegetation

The friction factor caused by the presence of vegetation is now obtained by substituting Eqs (10 and 12) into Eq. (7) to yield

13 $${f}_{v}=4{C}_{d}{(\frac{{U}_{v}}{{U}_{b}})}^{2}.$$

This expression is similar to a prior result given by^9^

14 $${f}_{v,poggi}=4{(\frac{{L}_{c}}{{h}_{v}})}^{-1}{[1+(1-\frac{1}{\alpha })\frac{{\rm{\Delta }}U}{{U}_{v}}]}^{-2},$$

derived from a different set of assumptions. To illustrate the similarities between the two expressions, consider the canopy-level adjustment length scale ${L}_{c}={({C}_{d}mD)}^{-1}$ as defined by the aforementioned study and ${\rm{\Delta }}U={U}_{s}-{U}_{v}$ to be the velocity difference between the vegetation zone *U*_*v*_ and the free water zone *U*_*s*_. Equation (14) can be arranged to read ${f}_{v,poggi}=4{C}_{d}mD{h}_{v}\,{({U}_{v}/{U}_{b})}^{2}$, where *C*_*d*_ is a local drag coefficient per unit frontal area (as shown next). More relevant here is that Eq. (13) allows for multiple effects to be conveniently included in a dimensionless *C*_*d*_ (instead of a dimensional *L*_*c*_) and *U*_*v*_/*U*_*b*_. Before doing so, it is to be noted that when submergence $\alpha ={h}_{v}/{h}_{w}\ll 1$^44^, ${U}_{v}\sim {u}_{\ast }$ and the conventional expression for friction factor is recovered from Eq. (13). To show explicitly similarities and differences between Eqs (13 and 14), the force balance for submerged conditions is rewritten per unit ground area as

15 $$\rho g{S}_{f}{R}_{h}={\tau }_{v,poggi},$$

so that

16 $$\rho g{S}_{f}\,[{h}_{w}(1-\alpha \varphi )]={C}_{d}\,(mD{h}_{v})\frac{\rho {U}_{v}^{2}}{2},$$

where *C*_*d*_ is a local drag coefficient per unit frontal area as before. Equation (14) further assumes the hydraulic radius to be the flow depth, or ${R}_{h}={h}_{w}(1-\alpha \varphi )\approx {h}_{w}$. Using this definition of ${\tau }_{v,poggi}={C}_{d}mD{h}_{v}\rho {U}_{v}^{2}/2$ and inserting this definition into the definition of $f=8\tau /(\rho {U}_{b}^{2})$ yields ${f}_{v,poggi}=4{C}_{d}mD{h}_{v}\,{({U}_{v}/{U}_{b})}^{2}$. However, when using a vegetation-related hydraulic radius ${R}_{v}={V}_{w}/{A}_{v}={h}_{w}(1-\alpha \varphi )/(mD{h}_{v})$ instead of *R*_*h*_ as proposed here, Eq. (16) can be transformed as

17 $$\rho g{S}_{f}\,[\frac{{h}_{w}(1-\alpha \varphi )}{mD{h}_{v}}]={C}_{d}\frac{\rho {U}_{v}^{2}}{2},$$

and the expression

18 $$\rho g{S}_{f}{R}_{v}={\tau }_{v,present},$$

is recovered where *C*_*d*_ remains a local drag coefficient per unit frontal area (as before). Naturally, when setting ${\tau }_{v,present}={C}_{d}\rho {U}_{v}^{2}/2$ into the definition of $f=8\tau /(\rho {U}_{b}^{2})$, the outcome ${f}_{v,present}=4{C}_{d}\,{({U}_{v}/{U}_{b})}^{2}$ of Eq. (13) is recovered. It can be surmised that different *R*_*h*_ definitions yield expressions for *f* that appear to be different. However, not withstanding these apparent differences, the *C*_*d*_ remains a local drag coefficient per unit frontal area in all of them.

Because *C*_*d*_ is to be related to a local Reynolds number, several possibilities are now reviewed for defining a local Reynolds number in the presence of vegetation. Etminan *et al*.^55^ compared Reynolds numbers using various characteristic velocity scales (*U*_*v*,*b*_, *U*_*v*,*p*_, *U*_*v*,*c*_ and *U*_*v*,*s*_) with a fixed characteristic length scale *D* giving *Re*_*v*,*b*_, *Re*_*v*,*p*_, *Re*_*v*,*c*_ and *Re*_*v*,*s*_. The aforementioned work then showed that typical *C*_*d*_ formulation for a single cylinder can be employed to predict a drag coefficient for staggered vegetation when using *U*_*v*,*c*_ as the reference velocity to form ${R}{{e}}_{v,c}={U}_{v,c}D/\nu$ and resulting in a best-fit expression applicable for $0 < {R}{{e}}_{v,c} < 6000$ given as

19 $${C}_{d,ELG}=1+10{R}{{e}}_{v,c}^{-2/3},$$

where suffix ‘EGL’ denotes the first letter of the surname of each author in Etminan *et al*.^55^. Here, vegetation effects are introduced in both velocity and length scales by selecting the pore velocity (*U*_*v*_ or *U*_*b*_) and the vegetation-related hydraulic radius *R*_*v*_ for defining a Reynolds number. For emergent canopies, only the canopy layer exists and the Reynolds number is labeled as *Re*_*v*,*v*_ (in the suffix, the first letter ‘*v*’ is vegetation layer, and second letter ‘*v*’ denotes the characteristic length in the Reynolds number to be the vegetation-related hydraulic radius) given as

20 $$R{e}_{v,v}=\frac{{U}_{v}{R}_{v}}{\nu },$$

where *R*_*v*_ is the vegetation-related hydraulic radius as before, given as

21 $${R}_{v}=\frac{\pi D(1-\varphi )}{4\varphi }.$$

For a submerged vegetation, the Reynolds number *Re*_*b*,*v*_ can also be defined as (in the suffix the first letter ‘*b*’ is bulk flow, and second letter ‘*v*’ denotes the characteristic length in the Reynolds number to be vegetation-related hydraulic radius)

22 $$R{e}_{b,v}=\frac{{U}_{b}{R}_{v}}{\nu },$$

where *R*_*v*_ is, as before, the vegetation-related hydraulic radius now given as

23 $${R}_{v}=\frac{\pi D\,(1-\alpha \varphi )}{4\alpha \varphi }.$$

## Friction Factor for Flow Through Emergent Vegetation

For emergent vegetation in flow (*h*_*v*_ > *h*_*w*_)

24 $${f}_{v}=4{C}_{d},$$

Cheng’s result^36^ for emergent vegetated flow is recovered. In the aforementioned study, *f* was shown to be a monotonically decreasing function of Reynolds number *Re*_*v*,*v*_ consistent with several studies^29,39,59,62,63,67,68^. The relation between *f* and Reynolds number is analyzed using several experiments described next.

### Laboratory experiments

#### Present study

Flume experiments were conducted in a 10 m long and *B* = 0.3 m wide glass flume at the State Key Laboratory of Water Resources and Hydro-power Engineering Science at Wuhan University in China. The flume bed was set flat and the vegetation stems were arranged in linear configuration to ensure a locally uniform resistance. The vegetation was composed of cylinders with diameter *D* = 8 mm and height *h*_*v*_ = 250 mm. The cylindrical vegetation array was then positioned on a 10 mm thick plastic board covered with holes to allow for variable cylinder density variation (or *m*). Let ${\varphi }_{board}={m}_{0}\pi {D}^{2}/4$ be the fractional area covered by holes on the bare board with *m*_0_ being the number of holes on the bare board per unit board area being used for each run. Eight vegetation densities were then used, labeled Runs A to H, with $\varphi$ = 0.419, 0.291, 0.206, 0.163, 0.073, 0.041, 0.018 and 0.010, respectively. For all the runs, steady non-uniform flow was conducted with constant flow rate $Q=0.00384\,\,{{\rm{m}}}^{3}\,{s}^{-1}$.

A local *C*_*d*,*local*_ – *Re* function in steady non-uniform flow was derived and shown to be parabolic in shape (i.e. not monotonic) within the vegetation zone for a given vegetation density. Here, the focus is on the averaged drag for the entire vegetation zone, which can be expressed as

25 $${C}_{d}={\int }_{0}^{1}\,{C}_{d,local}({x}^{+})\,d{x}^{+}$$

where *C*_*d*,*local*_ is a local drag coefficient that varies in the streamwise direction along the vegetation zone, the normalized distance is ${x}^{+}=x/{L}_{veg}$ along the streamwise direction, and *L*_*veg*_ is the streamwise length of the vegetation zone. A summary of the data used here is given in Table 1 and the details are shown in Supplementary Information (Table S1). Also, further details about the experimental setup can be found elsewhere^51^.

**Table 1:** Summary for emergent vegetation in flow.

Authors	*ϕ* (%)	*D* (m)	*U*_*v*_ (m/s)	*Re*_*v*, *v*_	*C*_*d*_
Present study	1.0–41.9	0.008	0.144–0.306	1256–190530	1.04–1.54
Ishikawa *et al*.^59^	0.3–3.2	0.004–0.0064	0.165–0.908	24883–904213	0.56–1.29
Tanino and Nepf^62^	9.0–35.0	0.0064	0.004–0.105	54–5266	1.54–9.65

Two other published data sets from Ishikawa *et al*.^59^ and Tanino *et al*.^62^ are used here and are briefly summarized in Table 1.

#### Experiments conducted by Ishikawa *et al*

The first data set was generated from a 15 m long, 0.3 m wide, and 0.3 m deep channel using two types of steel cylinders with diameters 0.004 m and 0.0064 m, and a height of 0.2 m as described elsewhere^59^. The cylinder spacing was uniform in each case (=0.0632 m and 0.0316 m, respectively). The flow was nearly uniform and the steel canopy array was arranged in staggered configuration. The hydraulic parameters required here are given in Supplementary Information (Table S1).

#### Experiments conducted by Tanino *et al*

Tanino *et al*.^62^ conducted their experiments in two Plexiglas recirculating flumes. Cylindrical maple dowels with diameter *D* = 0.0064 m were used as laboratory models for vegetation. The vegetation array fractional volume varied as $\varphi$ = 0.091, 015, 0.20, 0.27, 0.35. The following data points (shown in Supplementary Information Table S1) were tracked from their best-fitting curve to yield an approximate expression

26 $${C}_{d,tn}=2\,(\frac{{T}_{1}}{{R}{{e}}_{d}}+{T}_{2}),$$

where suffix ‘*tn*’ denotes the expression of Tanino *et al*.^62^, *T*_1_ and *T*_2_ are obtained from linear regression. The *Re*_*v*,*d*_ in their formulation was based on a stem Reynolds number given as

27 $$R{e}_{v,d}=\frac{{U}_{v}D}{\nu },$$

which uses *D* as characteristic length for their Reynolds number.

### Empirical expressions

For flow through emergent vegetation, a large synthesis study proposed a *C*_*d*_ − *Re* expression given as^36^

28 $${C}_{d,cheng}=\frac{50}{R{e}_{v,v}}+0.7[1-\exp (\,-\,\frac{R{e}_{v,v}}{15000})].$$

The linkage between vegetation-array and stem related Reynolds number is

29 $$R{e}_{v,v}=\frac{{R}_{v}}{D}R{e}_{v,d}=\frac{\pi \,(1-\varphi )}{4\varphi }R{e}_{v,d}\mathrm{.}$$

Here, a simplified expression that summarizes all the aforementioned expressions may be expressed as a function of *Re*_*v*,*v*_ (desirable for the purposes of the work here) as

30 $${C}_{d}=0.819+\frac{58.5}{\sqrt{R{e}_{v,v}}}\mathrm{.}$$

**Figure 2:**
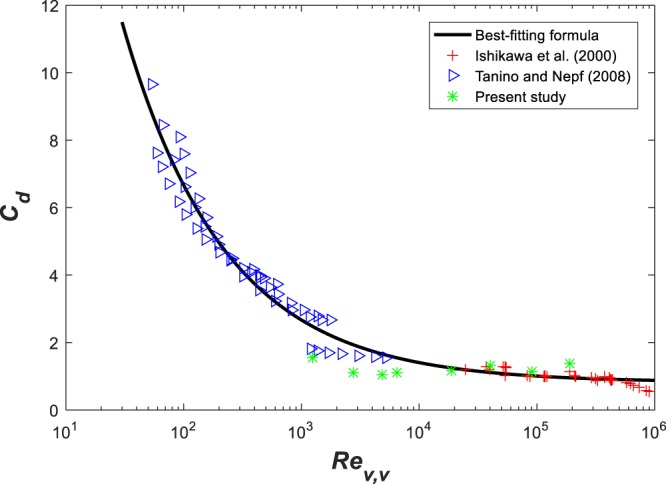
Best-fit expression for the local *C*_*d*_ − *Re*_*v*,*v*_ relation for the emergent vegetation case.

This summary expression is shown in Fig. 2, which an be re-arranged to be related to the more commonly reported *Re*_*v*,*d*_ as

31 $${C}_{d}=0.819+\frac{58.5}{\sqrt{\frac{\pi \,(1-\varphi )}{4\varphi }R{e}_{v,d}}}\mathrm{.}$$

Based on this drag coefficient, the *f* for emergent vegetation is given as a function of *Re*_*v*,*v*_

32 $${f}_{v,emergent}=3.276+\frac{234}{\sqrt{{R}{{e}}_{v,v}}},$$

or as a function of *Re*_*v*,*d*_

33 $${f}_{v,emergent}=3.276+\frac{234}{\sqrt{\frac{\pi \,(1-\varphi )}{4\varphi }R{e}_{v,d}}}\mathrm{.}$$

## Friction Factor for Flow Through Submerged Vegetation

### Laboratory experiments

For submerged vegetated flow, data were used from several published experiments and classified as either rigid or flexible. Data for the rigid vegetation flow are described elsewhere^29,31,38,57,69–76^. Data for flexible vegetation are also described elsewhere^24,69,76–81^. A summary of all the data points are in Table 2 and the details can be found in Supplementary Information (Tables S2 and S3).

**Table 2:** Summary for submerged vegetation flow.

Vegetation type	Authors	*Q* (m^3^ *s*^−1^)	*B* (m)	*H* (m)	*S*_*o*_ (%)	*ϕ* (%)	*D* (m)	*h*_*v*_ (m)	*m* (stems m^−2^)
Rigid vegetation	Dunn^69^	0.046–0.181	0.91	0.164–0.391	0.36–1.61	0.14–1.23	0.006	0.118	43–387
	Ghisalberti and Nepf^31^	0.002–0.014	0.38	0.467	0.0002–0.01	1.26–4.02	0.006	0.138–0.139	391–1250
	Liu *et al*.^29^	0.011	0.30	0.087–0.119	0.30	0.31–1.57	0.006	0.076	97–496
	López and García^70^	0.046–0.181	0.91	0.164–0.391	0.36–1.61	0.14–1.24	0.006	0.12	42–384
	Meijer^71^	0.866–8.98	3.00	0.990–2.500	0.055–0.205	0.32–1.29	0.008	0.45–1.5	64–256
	Murphy *et al*.^72^	0.002–0.014	0.38	0.088–0.467	0.0003–0.1340	1.18–3.77	0.006	0.070–0.140	417–1333
	Nezu and Sanjou^38^	0.003–0.008	0.40	0.063–0.200	0.0196–0.1553	4.76–18.48	0.008	0.050	947–3676
	Poggi *et al*.^57^	0.162	0.90	0.600	0.004–0.0320	0.08–1.35	0.004	0.12	67–1072
	Shimizu *et al*.^73^	0.002–0.016	0.40–0.50	0.050–0.106	0.0660–0.7000	0.44–0.79	0.01–0.02	0.041–0.046	2501–9995
	Stone and Shen^74^	0.002–0.065	0.45	0.151–0.314	0.009–4.400	0.55–6.11	0.003–0.013	0.124	166–692
	Yan^75^	0.014–0.038	0.42	0.120–0.300	0.065–1.280	1.41–5.65	0.006	0.06	500–2000
	Yang and Choi^76^	0.008–0.011	0.45	0.075	0.141–0.269	0.44	0.002	0.035	1400
Flexible vegetation	Dunn^69^	0.078–0.180	0.91	0.230–0.367	0.36–1.01	0.14–1.23	0.006	0.097–0.161	43–388
	Yang and Choi^76^	0.008–0.011	0.45	0.055–0.110	0.07–0.361	0.44	0.002	0.023–0.034	1400
	Kubrak *et al*.^77^	0.027–0.075	0.58	0.180–0.266	0.87–1.74	0.13–0.53	0.001	0.131–0.164	2500–10000
	Okamoto and Nezu^78^	0.006–0.032	0.40	0.150–0.315	0.019–0.2409	4.78	0.008	0.03–0.096	951
	Järvelä^79^	0.040–0.143	1.10	0.306–0.707	0.02–0.36	7.39	0.003	0.155–0.260	12000
	Carollo *et al*.^80^	0.027–0.189	0.60	0.119–0.277	0.2–1.0	2.2–3.46	0.001	0.031–0.082	28000–44000
	Ciraolo and Ferreri^24^	0.027–0.177	0.77	0.150–0.478	0.008–0.85	2.04	0.005	0.063–0.290	1037
	Kouwen *et al*.^81^	0.003–0.142	0.61	0.149–0.400	0.05–1.0	9.82	0.005	0.050–0.100	5000

### Determination of depth-averaged velocity in the vegetation and surface layers

**Figure 3:**
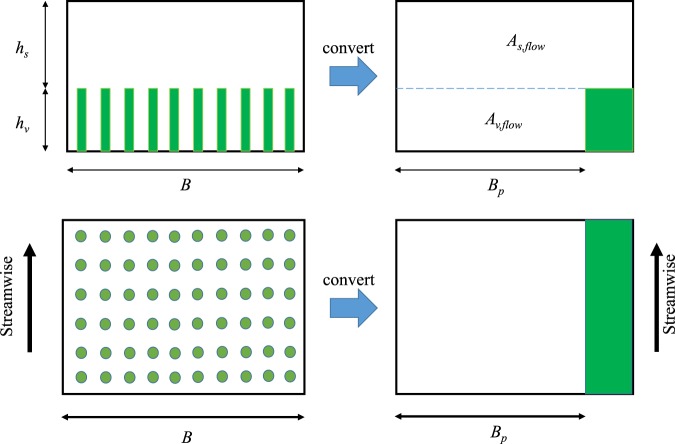
Side and top views of submerged vegetation flow and the definition of effective width *B*_*p*_ from the geometric width *B*.

In the vegetation layer (VL), the depth-averaged velocity can be derived based on the force balance between vegetation drag and flow gravity in streamwise direction

34 $${U}_{v}=\sqrt{\frac{2g{S}_{o}\,(1-\alpha \varphi )}{{C}_{d}mD\alpha }},$$

where *C*_*d*_ is calculated from Eqs (30 or 31), indicating iterations are needed to obtain the velocity and drag coefficient. Moreover, the effective width of the canopy layer for the pore velocity ${B}_{p} < B$ is briefly discussed. Figure 3 shows side and top views of the flume with cylindrical vegetation, where the green zone indicates the area occupied by vegetation. The effective width *B*_*p*_ is expressed as ${B}_{p}=B\,(1-\varphi )$.

For surface layer (SL), the depth-averaged velocity can be determined by the linkage between the vegetation-layer velocity *U*_*v*_ and the bulk velocity *U*_*b*_. The bulk velocity is defined by the total discharge *Q* to the effective cross-sectional area and given as (pore velocity for bulk flow)

35 $${U}_{b}=\frac{Q}{{A}_{e}}=\frac{Q}{{h}_{s}B+{h}_{v}B\,(1-\varphi )}.$$

From the continuity equation,

36 $$Q={Q}_{s}+{Q}_{v}={U}_{s}{A}_{s,flow}+{U}_{v}{A}_{v,flow},$$

where *Q*_*s*_, *A*_*s*,*flow*_ are the flow rate and the effective cross-sectional area in the surface layer, and *Q*_*v*_, *A*_*v*,*flow*_ are the flow rate and the effective cross-sectional area in the vegetation layer. The relation between *U*_*b*_, *U*_*s*_ and *U*_*v*_ is derived as

37 $${U}_{b}=\frac{{U}_{s}{h}_{s}+{U}_{v}{h}_{v}\,(1-\varphi )}{{h}_{s}+{h}_{v}\,(1-\varphi )}.$$

Arranging the above equation, the bulk velocity is given by

38 $${U}_{b}=\frac{1-\alpha }{1-\alpha \varphi }{U}_{s}+\frac{\alpha (1-\varphi )}{1-\alpha \varphi }{U}_{v},$$

then the depth-averaged velocity of surface layer gives

39 $${U}_{s}=\frac{(1-\alpha \varphi )}{1-\alpha }{U}_{b}-\frac{\alpha \,(1-\varphi )}{1-\alpha }{U}_{v}.$$

### Methods to determine friction factor in the submerged cases

**Figure 4:**
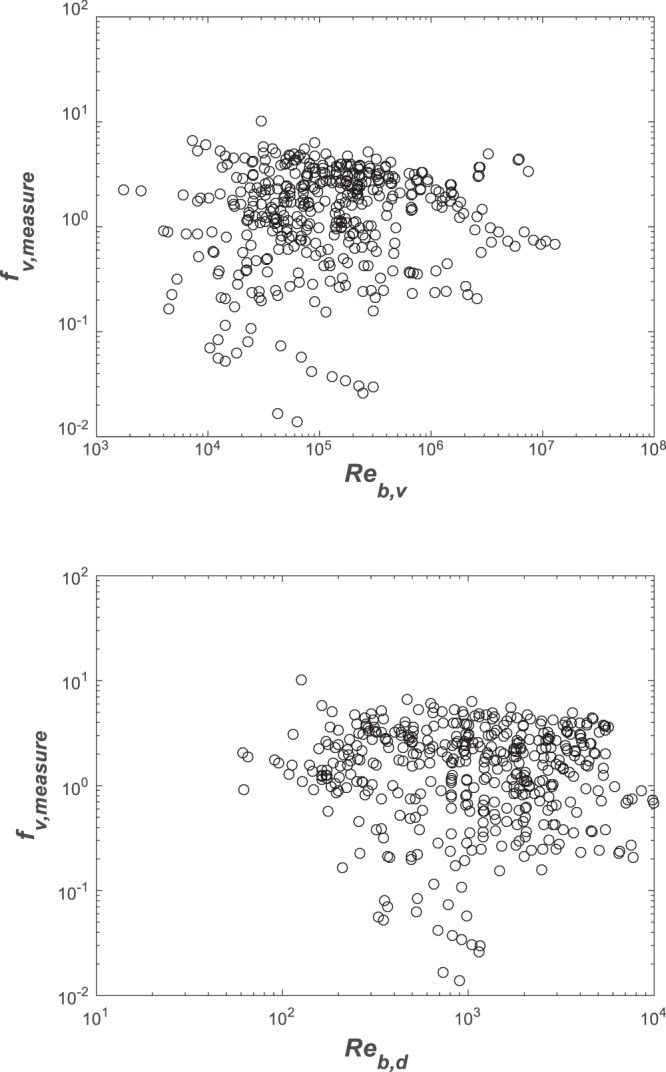
Measured variations in *f*_*v*_ with Reynolds number *Re*_*b*,*v*_ and *Re*_*b*,*d*_ illustrating no obvious trends when experiments are combined.

The measured friction *f*_*v*,*measure*_ can be obtained from *U*_*v*_ (using Eq. 34), *U*_*b*_ (using Eq. 35) and *C*_*d*_ (using Eqs 30 or 31) when setting ${f}_{v}=4{C}_{d}\,{({U}_{v}/{U}_{b})}^{2}$. All data points and their variation with the Reynolds number *Re*_*b*,*v*_ or *Re*_*b*,*d*_ are given in Fig. 4. The results suggest no obvious trends between measured *f*_*v*_ and estimated *Re*_*b*,*v*_ or *Re*_*b*,*d*_.

The absence of a unique relation between *f*_*v*_ and Reynolds number requires further inquiry. The key to calculating *f*_*v*_ is *U*_*v*_/*U*_*b*_. Previous studies^9^ showed that *f*_*v*_ is linked to two dimensionless groups: $\alpha ={h}_{v}/{h}_{w}$ and $\eta ={h}_{v}/{L}_{c}$. As mentioned before, the adjustment canopy length scale is defined as

40 $${L}_{c}=\frac{1}{{C}_{d}mD}.$$

The aforementioned study^9^ did show a nonlinear increase in *f*_*v*_ with increasing *α* at a given $\eta$, and a nonlinear increase in *f*_*v*_ with increasing $\eta$ at a given *α*. Based on these results, we propose a 3-parameter mathematical function to describe this behavior without focusing on the detailed mean velocity profile in each zone. This 3-parameter function is given as

41 $${(\frac{{U}_{v}}{{U}_{b}})}^{2}=\frac{{p}_{1}{\alpha }^{2}}{{p}_{2}\alpha +{p}_{3}\eta }$$

where *p*_1_, *p*_2_, *p*_3_ are constants to be determined from regression analysis. The corresponding friction *f*_*v*_ can now be expressed as

42 $${f}_{v,present}=4{C}_{d}(\frac{{p}_{1}{\alpha }^{2}}{{p}_{2}\alpha +{p}_{3}\eta }).$$

**Figure 5:**
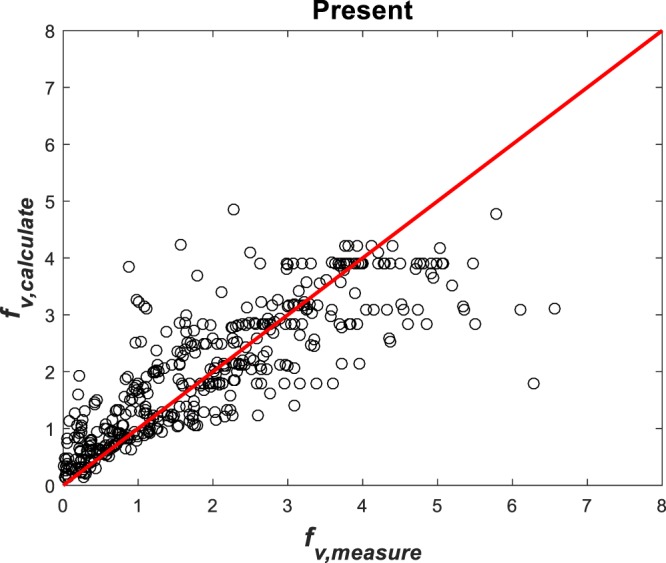
Comparison between measured and predicted *f*_*v*_ for all the data sets combined.

This equation shows *f*_*v*_ increases with increasing submergence $\alpha ={h}_{v}/{h}_{w}$ when other parameters are held constant, and increases with increasing $\eta ={h}_{v}/{L}_{c}$ when other parameters are held constant. With a local *C*_*d*_ determined from Eq. (31), parameters ${p}_{1}=1.198$, ${p}_{2}=0.681$, ${p}_{3}=0.416$ where determined using non-linear regression with a correlation coefficient ${R}_{cc}=0.803$ as shown in Fig. 5.

### Friction factor derived from previous studies

Some studies report expressions for the velocity within the vegetated zone, which can also be used to obtain a friction factor. A summary of these expressions are briefly reviewed. Stone *et al*.^74^ estimated the bulk velocity as

43 $${U}_{b}=1.385\,(\frac{1}{\alpha }\sqrt{\frac{\pi }{4\varphi }}-1)\sqrt{gD{S}_{f}}.$$

Baptist *et al*.^41^ report a bulk velocity as

44 $${U}_{b}=[\sqrt{\frac{1}{g/{C}_{b}^{2}+2{C}_{d}\varphi {h}_{v}/(\pi D)}}+\frac{5}{2}\,\mathrm{ln}\,(\frac{1}{\alpha })]\sqrt{g{h}_{w}{S}_{f}},$$

where *C*_*d*_ is the bed-related Chezy coefficient (=60 m^1/2^ s^−1^ for smooth bed).

Huthoff *et al*.^28^ proposed a

45 $${U}_{b}=[(1-\alpha )\,{(\frac{{h}_{s}}{D\sqrt{\pi /(4\varphi )}-D})}^{\frac{2}{3}[1-{\alpha }^{5}]}+\sqrt{\alpha }]\sqrt{\frac{\pi gD{S}_{f}}{2{C}_{d}\varphi }}.$$

Yang *et al*.^82^ showed a bulk velocity given as

46 $${U}_{b}=\sqrt{\frac{\pi gD{S}_{f}}{2{C}_{d}\alpha \varphi }}+\frac{{C}_{u}\sqrt{g{h}_{s}{S}_{f}}}{\kappa }(\mathrm{ln}\,\frac{1}{\alpha }+\alpha -1),$$

where ${C}_{u}=1$ for $4\varphi /(\pi D)\le 5$ and ${C}_{u}=2$ for $4\varphi /(\pi D) > 5$.

Cheng *et al*.^83^ derived the representative roughness height of the vegetation for the surface layer and proposed

47 $${U}_{b}=[\sqrt{\frac{\pi D\,{(1-\varphi )}^{3}}{2{C}_{d}\varphi {h}_{v}}}{\alpha }^{3/2}+4.54\,{(\frac{{h}_{s}}{D}\frac{1-\varphi }{\varphi })}^{1/16}{(1-\alpha )}^{3/2}]\sqrt{g{h}_{w}{S}_{f}}.$$

Katul *et al*.^44^ proposed an analytical model for *U*_*b*_ linked to three canonical length scales (*L*_*c*_, *h*_*v*_, *h*_*w*_) and is given as

48 $${U}_{b}=\alpha {U}_{KCL}+(1-\alpha )\,{U}_{KSL},$$

where *U*_*KCL*_, *U*_*KSL*_ are layer-averaged velocity for the canopy and surface layers, respectively, given as

49 $${U}_{KCL}=\frac{2\beta }{\eta }[1-\exp (\,-\,\frac{\eta }{2{\beta }^{2}})]\sqrt{g{h}_{s}{S}_{f}},$$

and

50 $${U}_{KSL}=\frac{1}{\kappa }\,\{\,-\,1+\,{\rm{l}}{\rm{n}}\,[{(\frac{{h}_{w}-D}{{z}_{0}})}^{({h}_{w}-D)/{h}_{s}}{(\frac{{h}_{v}-D}{{z}_{0}})}^{-({h}_{v}-D)/{h}_{s}}]\}\sqrt{g{h}_{s}{S}_{f}},$$

where $\beta =\,{\min }(0.135\sqrt{mD},0.33)$ is a momentum absorption coefficient, and *z*_0_ is given by

51 $${z}_{0}=\frac{2\beta {L}_{c}}{\kappa }\exp (\,-\,\frac{\kappa }{\beta }).$$

With these mean velocity profile formulations, friction factor can be estimated. The bulk velocity of the vegetation layer is determined using Eq. (34) and the velocity across *h*_*w*_ is calculated based on Eqs (43 to 48).

### Comparison between measured and modeled friction factor using published bulk velocities

**Figure 6:**
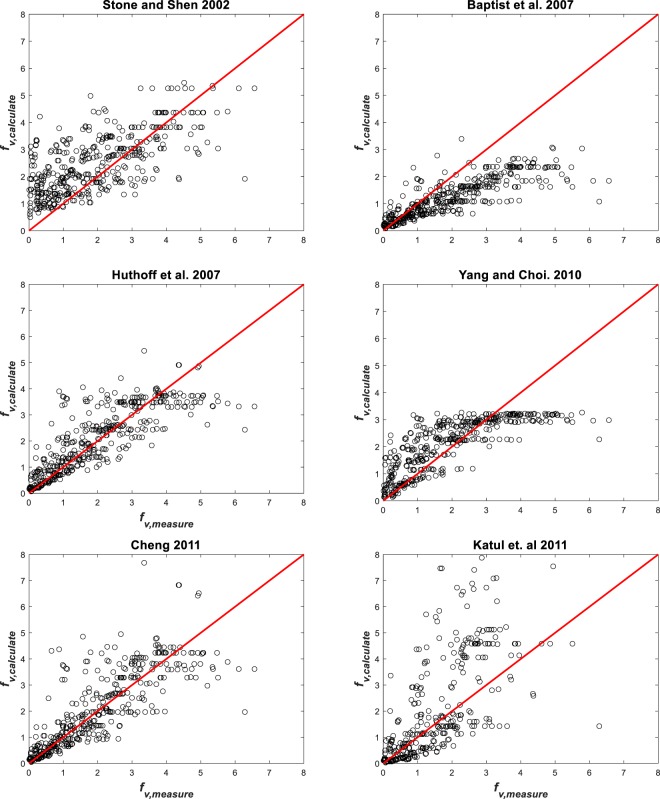
Comparison between measured and modeled friction factor using different methods.

The different methods are now used to predict *f*_*v*_ and are then compared with measured *f*_*v*_ in Fig. 6. To assess the agreement between prediction and measurement, *R*_*cc*_ is computed and reported in Table 3. Figure 6 suggests that the present model performs ‘no worse’ than other (more elaborate) methods. In many cases, it even outperforms prior methods.

**Table 3:** Comparison between measured and modeled friction factor by different formulations.

Authors	Correlation coefficient *R*_*cc*_
Present study	0.803
Stone and Shen^74^	0.654
Baptist *et al*.^41^	0.806
Huthoff *et al*.^28^	0.790
Yang and Choi^82^	0.758
Cheng^83^	0.783
Katul *et al*.^44^	0.637

## Discussion and Conclusion

Equation ${f}_{v}=4{C}_{d}\,{({U}_{v}/{U}_{b})}^{2}$ encodes much of the recent developments about vegetation effects on local drag (e.g. blockage and sheltering, weak Reynolds number dependence, etc) and their up-scaled contribution on *f*_*v*_ via *U*_*v*_/*U*_*b*_. These effects are now briefly discussed. For isolated cylinders, the local *C*_*d*_ can be determined from Cheng^43^

52 $${C}_{d,iso}=11{R}{{e}}_{v,d}^{-0.75}+0.9{{\rm{\Gamma }}}_{1}\,({R}{{e}}_{v,d})+1.2{{\rm{\Gamma }}}_{2}\,({R}{{e}}_{v,d}),$$

where

53 $${{\rm{\Gamma }}}_{1}\,({R}{{e}}_{v,d})=1-\exp \,(-\frac{1000}{{R}{{e}}_{v,d}}),$$

and

54 $${{\rm{\Gamma }}}_{2}\,({R}{{e}}_{v,d})=1-\exp \,[-{(\frac{{R}{{e}}_{v,d}}{4500})}^{0.7}].$$

Many studies found that *C*_*d*_ in a vegetated array differs from *C*_*d*,*iso*_. When only focusing on variations in vegetation density, *C*_*d*_ appears to increase^62,68^ and then decrease^60,84^ with increasing vegetation density^55^. In the present study, we go beyond vegetation density and introduce all the key vegetation effects in a Reynolds number ${R}{{e}}_{v,v}={U}_{v}{R}_{v}/\nu$ formed by *U*_*v*_ and *R*_*v*_. These velocity and length scales contain the vegetation density $\varphi$ and appear to collapse the experiments onto a single curve.

To accommodate the mechanics of sheltering, delayed separation and blockage effects arising from an array of cylinders instead of isolated cylinders, *C*_*d*_ of Eq. (31) can be used. Sheltering effect indicates that some elements were located in the wake region of the upstream elements^85^, resulting in a lower velocity than their upstream counterparts and generate a lower drag compared with the isolated cylinder case. Delayed separation can be explained by the enhancement of the mean separation angle that is larger than that for the isolated cylinder, resulting in a decreasing drag coefficient (compared with the isolated cylinder). Both sheltering and delayed separation tend to reduce drag when compared to the isolated cylinder case. For the blockage effect, which leads to a local increase in the drag coefficient, it can by explained by two main factors^55^, one is that the velocity between cylinders is enhanced by the presence of the vegetation in flow. The other factor is reduced wake pressure^86^.

The *C*_*d*,*iso*_ is a local drag coefficient acting on a single stem per unit frontal area without considering the interaction among elements in a vegetation array. To illustrate the role of sheltering, delayed separation and blockage on *C*_*d*_, a ‘bifurcation’ type Reynolds number ${R}{{e}}_{v,d}^{+}$ may be introduced. When ${R}{{e}}_{v,d} < {R}{{e}}_{v,d}^{+}$, the viscous boundary layer formed around cylinders creates a slow-moving flow zone with a path smaller than the spacing between adjacent stem. This effect leads to an equivalent local drag coefficient by the array of cylinders to be larger than the one associated with isolated cylinders (i.e. a blockage effect). However, when ${R}{{e}}_{v,d} > {R}{{e}}_{v,d}^{+}$, vegetation stems become a new source of turbulent kinetic energy (wake production) spawning horizontal vortices resembling von Karman streets that grow in size and ‘fill’ the space between the stems. This effect is akin to a decreasing drag coefficient when compared with *C*_*d*,*iso*_ (i.e. sheltering effect). Blockage and sheltering effects can be distinguished by calculating

55 $$E=\frac{{C}_{d}}{{C}_{d,iso}}.$$

**Figure 7:**
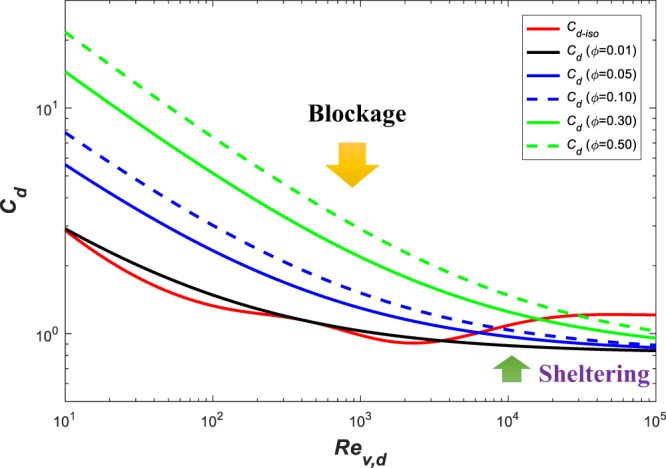
Comparison between local *C*_*d*,*iso*_ and *C*_*d*_ for different vegetation concentration.

When $E > 1$, blockage dominates and when $E < 1$, sheltering dominates *C*_*d*_^51^. Using Eqs (31 and 52) with different vegetation concentration ($\varphi$ = 0.01, 0.05, 0.1, 0.3 and 0.5), blockage and sheltering can be delineated for *Re*_*v*,*d*_ ranging from 10^1^ to 10^5^ in Fig. 7. As expected, *C*_*d*_ increases with increasing $\varphi$ for small $\varphi$. The threshold Reynolds number ${R}{{e}}_{v,d}^{+}$ also increases with increasing $\varphi$. For example, at ${R}{{e}}_{v,d}^{+}\approx 4000$, separation between blockage and sheltering occurs for $\varphi =0.01$. This threshold ${R}{{e}}_{v,d}^{+}$ becomes much larger, around 30000 for $\varphi =0.5$. For $\varphi =0.01$, when ${R}{{e}}_{v,d} < {R}{{e}}_{v,d}^{+}$, the *C*_*d*_ for isolated stems are comparable but not identical to their array counterpart.

To conclude, the expression of friction factor proposed here does accommodate blockage or sheltering through a local drag coefficient *C*_*d*_ (Eq. 31) and any distortions to the shape of the mean velocity profile (Eq. 41). Evaluated using numerous data sets covering a wide range of canopy morphology, densities and rigidity, the friction factor for emergent vegetation is shown to be proportional to the drag coefficient. This finding shows why the friction factor monotonically decreases with increasing Reynolds number for emergent vegetation. For submerged vegetation, the friction factor is shown to vary with submergence and adjustment length scale. Also, this variation is not monotonic with increasing Reynolds number.

## Supplementary information

Supplementary
