# Supplementary material for: Friction factor for turbulent open channel flow covered by vegetation
Source: Sci Rep. 2019 Mar 26;9:5178. doi: 10.1038/s41598-019-41477-7 (PMC6435652; doi:10.1038/s41598-019-41477-7)
Supplement: Supplementary file 1 — Supplementary [file 41598_2019_41477_MOESM1_ESM.pdf]

Supplementary Information for

Friction factor for turbulent open channel flow covered by vegetation

Wei-Jie Wang [1,2], Wen-Qi Peng [1,2], Wen-Xin Huai [3], Gabriel G. Katul [4,5],

Xiao-Bo Liu [1,2], Xiao-Dong Qu [1,2], Fei Dong [1,2]

[1] State Key Laboratory of Simulation and Regulation of Water Cycle in River Basin, China Institute of Water Resources and Hydropower Research, Beijing 100038, China.

[2] Department of Water Environment, China Institute of Water Resources and Hydropower Research, Beijing 100038, China.

[3] State Key Laboratory of Water Resources and Hydropower Engineering Science, Wuhan University, Wuhan, Hubei 430072, China.

[4] Nicholas School of the Environment, Duke University, Durham, North Carolina 27708, USA.

[5] Department of Civil and Environmental Engineering, Duke University, Durham, North Carolina 27708, USA.

Contents of this file

Tables S1 to S3

Introduction

Experimental data of vegetated flow used in this paper were given in Tables S1 to S3.

Table S1. Experimental data of flow through emergent vegetation

Authors	ϕ	D (m)	U_v (m/s)	$Re_{v,v}$	C_d
**Present study**	0.419	0.008	0.144	1256	1.54
	0.291	0.008	0.181	2773	1.11
	0.206	0.008	0.201	4856	1.04
	0.163	0.008	0.202	6506	1.11
	0.073	0.008	0.236	18859	1.17
	0.041	0.008	0.276	40512	1.31
	0.018	0.008	0.265	90761	1.12
	0.01	0.008	0.306	190530	1.36
**Ishikawa et al. [48]**	0.0081	0.0064	0.306	189338	1.15
	0.0081	0.0064	0.335	207514	1.01
	0.0081	0.0064	0.344	213123	0.98
	0.0081	0.0064	0.471	291303	0.96
	0.0081	0.0064	0.510	315942	0.94
	0.0081	0.0064	0.605	374429	0.95
	0.0081	0.0064	0.669	413893	0.95
	0.0081	0.0064	0.708	438496	0.90
	0.0081	0.0064	0.531	328544	0.86
	0.0322	0.0064	0.248	37454	1.29
	0.0322	0.0064	0.343	51717	1.29
	0.0322	0.0064	0.354	53438	1.29
	0.0322	0.0064	0.361	54461	1.26
	0.0322	0.0064	0.260	39324	1.21
	0.0322	0.0064	0.165	24883	1.19
	0.0031	0.004	0.380	377933	0.99
	0.0031	0.004	0.419	417518	0.89
	0.0031	0.004	0.434	431972	0.87
	0.0031	0.004	0.574	571350	0.80
	0.0031	0.004	0.630	627474	0.77
	0.0031	0.004	0.656	652718	0.73
	0.0031	0.004	0.739	735925	0.66
	0.0031	0.004	0.845	840845	0.58
	0.0031	0.004	0.908	904213	0.56
	0.0126	0.004	0.220	54102	1.01
	0.0126	0.004	0.332	81940	1.03
	0.0126	0.004	0.354	87298	0.99
	0.0126	0.004	0.478	117837	1.01
	0.0126	0.004	0.467	115123	0.98
	0.0126	0.004	0.493	121410	0.98

	0.0126	0.004	0.478	117824	0.99
**Tanino and Nepf [51]**	0.09	0.0064	0.105	5266	1.54
	0.09	0.0064	0.084	4229	1.56
	0.09	0.0064	0.061	3063	1.60
	0.09	0.0064	0.044	2190	1.65
	0.09	0.0064	0.034	1714	1.70
	0.09	0.0064	0.027	1376	1.76
	0.09	0.0064	0.024	1194	1.80
	0.15	0.0064	0.062	1760	2.66
	0.15	0.0064	0.048	1360	2.79
	0.15	0.0064	0.036	1037	2.98
	0.15	0.0064	0.029	812	3.17
	0.15	0.0064	0.022	635	3.43
	0.15	0.0064	0.019	537	3.64
	0.15	0.0064	0.016	443	3.94
	0.15	0.0064	0.014	389	4.17
	0.2	0.0064	0.074	1488	2.66
	0.2	0.0064	0.058	1165	2.77
	0.2	0.0064	0.041	822	2.98
	0.2	0.0064	0.030	595	3.21
	0.2	0.0064	0.022	437	3.55
	0.2	0.0064	0.016	321	3.98
	0.2	0.0064	0.012	248	4.46
	0.2	0.0064	0.009	189	5.14
	0.2	0.0064	0.008	156	5.71
	0.2	0.0064	0.007	133	6.28
	0.2	0.0064	0.006	113	7.04
	0.2	0.0064	0.005	100	7.60
	0.2	0.0064	0.005	92	8.10
	0.27	0.0064	0.045	616	3.72
	0.27	0.0064	0.036	482	3.89
	0.27	0.0064	0.027	373	4.10
	0.27	0.0064	0.019	260	4.48
	0.27	0.0064	0.015	198	4.92
	0.27	0.0064	0.011	151	5.45
	0.27	0.0064	0.009	123	5.99
	0.27	0.0064	0.007	101	6.60
	0.27	0.0064	0.006	82	7.41
	0.27	0.0064	0.005	66	8.46
	0.27	0.0064	0.004	54	9.65
	0.35	0.0064	0.047	440	3.99

	0.35	0.0064	0.035	323	4.20
	0.35	0.0064	0.027	249	4.42
	0.35	0.0064	0.022	200	4.68
	0.35	0.0064	0.016	153	5.05
	0.35	0.0064	0.014	127	5.38
	0.35	0.0064	0.011	104	5.79
	0.35	0.0064	0.010	91	6.17
	0.35	0.0064	0.008	74	6.72
	0.35	0.0064	0.007	65	7.22
	0.35	0.0064	0.006	59	7.63

Table S2. Experimental data of flow through submerged rigid vegetation

Author	Q (m^3/s)	B (m)	h_w (m)	S_o	ϕ	D (m)	h_v (m)
Dunn [58]	0.17900	0.91	0.335	0.003600	0.00545	0.0064	0.118
	0.08800	0.91	0.229	0.003600	0.00545	0.0064	0.118
	0.04600	0.91	0.164	0.003600	0.00545	0.0064	0.118
	0.17800	0.91	0.276	0.007600	0.00545	0.0064	0.118
	0.09800	0.91	0.203	0.007600	0.00545	0.0064	0.118
	0.17800	0.91	0.267	0.003600	0.00136	0.0064	0.118
	0.09500	0.91	0.183	0.003600	0.00136	0.0064	0.118
	0.18000	0.91	0.391	0.003600	0.01226	0.0064	0.118
	0.05800	0.91	0.214	0.003600	0.01226	0.0064	0.118
	0.18000	0.91	0.265	0.016100	0.01226	0.0064	0.118
	0.17700	0.91	0.311	0.003600	0.00307	0.0064	0.118
	0.18100	0.91	0.233	0.010800	0.00307	0.0064	0.118
Ghisalberti and Nepf [18]	0.00480	0.38	0.467	0.000010	0.01258	0.0064	0.139
	0.00170	0.38	0.467	0.000002	0.01258	0.0064	0.139
	0.00740	0.38	0.467	0.000025	0.01708	0.0064	0.139
	0.00480	0.38	0.467	0.000012	0.01708	0.0064	0.139
	0.01430	0.38	0.467	0.000075	0.02011	0.0064	0.138
	0.00940	0.38	0.467	0.000032	0.02011	0.0064	0.138
	0.00480	0.38	0.467	0.000013	0.02011	0.0064	0.138
	0.01430	0.38	0.467	0.000100	0.04021	0.0064	0.138
	0.00940	0.38	0.467	0.000034	0.04021	0.0064	0.138
	0.00480	0.38	0.467	0.000013	0.04021	0.0064	0.138
	0.00170	0.38	0.467	0.000003	0.04021	0.0064	0.138
Liu et al. [16]	0.01140	0.30	0.097	0.003000	0.00614	0.0064	0.076
	0.01140	0.30	0.101	0.003000	0.01229	0.0064	0.076
	0.01140	0.30	0.087	0.003000	0.00307	0.0064	0.076
	0.01140	0.30	0.114	0.003000	0.01571	0.0064	0.076
	0.01140	0.30	0.115	0.003000	0.01571	0.0064	0.076

	0.01140	0.30	0.118	0.003000	0.01571	0.0064	0.076
	0.01140	0.30	0.119	0.003000	0.01571	0.0064	0.076
	0.01140	0.30	0.114	0.003000	0.01571	0.0064	0.076
	0.01140	0.30	0.119	0.003000	0.01571	0.0064	0.076
**López and García [59]**	0.17900	0.91	0.335	0.003600	0.00547	0.0064	0.120
	0.08800	0.91	0.229	0.003600	0.00547	0.0064	0.120
	0.04600	0.91	0.164	0.003600	0.00547	0.0064	0.120
	0.17800	0.91	0.276	0.007600	0.00547	0.0064	0.120
	0.09800	0.91	0.203	0.007600	0.00547	0.0064	0.120
	0.17800	0.91	0.267	0.003600	0.00135	0.0064	0.120
	0.09500	0.91	0.183	0.003600	0.00135	0.0064	0.120
	0.18000	0.91	0.391	0.003600	0.01235	0.0064	0.120
	0.05800	0.91	0.214	0.003600	0.01235	0.0064	0.120
	0.18000	0.91	0.265	0.016100	0.01235	0.0064	0.120
	0.17700	0.91	0.311	0.003600	0.00312	0.0064	0.120
	0.18100	0.91	0.233	0.011000	0.00312	0.0064	0.120
**Meijer [60]**	1.03950	3.00	1.980	0.001090	0.01287	0.0080	1.500
	1.39101	3.00	1.990	0.001800	0.01287	0.0080	1.500
	1.39284	3.00	2.190	0.000950	0.01287	0.0080	1.500
	1.56366	3.00	2.190	0.001250	0.01287	0.0080	1.500
	1.70610	3.00	2.350	0.000810	0.01287	0.0080	1.500
	2.35563	3.00	2.330	0.001540	0.01287	0.0080	1.500
	1.91250	3.00	2.500	0.000650	0.01287	0.0080	1.500
	2.72688	3.00	2.470	0.001430	0.01287	0.0080	1.500
	1.86327	3.00	2.010	0.001060	0.00322	0.0080	1.500
	2.52657	3.00	2.010	0.001930	0.00322	0.0080	1.500
	2.29020	3.00	2.200	0.001010	0.00322	0.0080	1.500
	3.07476	3.00	2.190	0.001880	0.00322	0.0080	1.500
	2.62260	3.00	2.350	0.000930	0.00322	0.0080	1.500
	3.45807	3.00	2.310	0.001870	0.00322	0.0080	1.500
	2.90904	3.00	2.480	0.000940	0.00322	0.0080	1.500
	3.94830	3.00	2.460	0.001780	0.00322	0.0080	1.500
	1.12344	3.00	1.510	0.001070	0.01287	0.0080	0.900
	1.61880	3.00	1.520	0.002040	0.01287	0.0080	0.900
	1.79733	3.00	1.810	0.000850	0.01287	0.0080	0.900
	2.55420	3.00	1.800	0.001650	0.01287	0.0080	0.900
	2.52681	3.00	2.090	0.000710	0.01287	0.0080	0.900
	3.61779	3.00	2.090	0.001380	0.01287	0.0080	0.900
	3.72000	3.00	2.480	0.000550	0.01287	0.0080	0.900
	5.96304	3.00	2.460	0.001490	0.01287	0.0080	0.900
	1.74858	3.00	1.510	0.001030	0.00322	0.0080	0.900
	2.52624	3.00	1.520	0.002050	0.00322	0.0080	0.900
	2.50323	3.00	1.810	0.000850	0.00322	0.0080	0.900

	3.52974	3.00	1.780	0.001800	0.00322	0.0080	0.900
	3.38310	3.00	2.100	0.000750	0.00322	0.0080	0.900
	4.72152	3.00	2.060	0.001640	0.00322	0.0080	0.900
	4.77945	3.00	2.470	0.000710	0.00322	0.0080	0.900
	6.68382	3.00	2.470	0.001430	0.00322	0.0080	0.900
	0.86598	3.00	1.020	0.000780	0.01287	0.0080	0.450
	1.30977	3.00	0.990	0.001640	0.01287	0.0080	0.450
	2.08833	3.00	1.510	0.000590	0.01287	0.0080	0.450
	3.06000	3.00	1.500	0.001380	0.01287	0.0080	0.450
	3.74220	3.00	1.980	0.000580	0.01287	0.0080	0.450
	5.62374	3.00	1.990	0.001420	0.01287	0.0080	0.450
	5.91876	3.00	2.460	0.000700	0.01287	0.0080	0.450
	7.17867	3.00	2.490	0.000900	0.01287	0.0080	0.450
	1.34028	3.00	1.020	0.000750	0.00322	0.0080	0.450
	1.98300	3.00	1.000	0.001870	0.00322	0.0080	0.450
	2.80800	3.00	1.500	0.000690	0.00322	0.0080	0.450
	4.77450	3.00	1.500	0.001990	0.00322	0.0080	0.450
	5.73000	3.00	2.000	0.000990	0.00322	0.0080	0.450
	7.31400	3.00	2.000	0.001590	0.00322	0.0080	0.450
	6.56952	3.00	2.480	0.000630	0.00322	0.0080	0.450
	8.97966	3.00	2.410	0.001270	0.00322	0.0080	0.450
**Murphy et al. [61]**	0.00480	0.38	0.467	0.000010	0.01179	0.0060	0.140
	0.00740	0.38	0.467	0.000025	0.01603	0.0060	0.140
	0.00480	0.38	0.467	0.000012	0.01603	0.0060	0.140
	0.01430	0.38	0.467	0.000075	0.01886	0.0060	0.140
	0.00480	0.38	0.467	0.000013	0.01886	0.0060	0.140
	0.01430	0.38	0.467	0.000100	0.03769	0.0060	0.140
	0.00940	0.38	0.467	0.000034	0.03769	0.0060	0.140
	0.00170	0.38	0.298	0.000003	0.01179	0.0060	0.070
	0.00940	0.38	0.298	0.000080	0.01179	0.0060	0.070
	0.00480	0.38	0.298	0.000024	0.01179	0.0060	0.070
	0.00170	0.38	0.236	0.000011	0.01179	0.0060	0.070
	0.00940	0.38	0.236	0.000116	0.01179	0.0060	0.070
	0.00480	0.38	0.236	0.000043	0.01179	0.0060	0.070
	0.00170	0.38	0.140	0.000017	0.01179	0.0060	0.070
	0.00940	0.38	0.140	0.000487	0.01179	0.0060	0.070
	0.00480	0.38	0.140	0.000301	0.01179	0.0060	0.070
	0.00170	0.38	0.105	0.000124	0.01179	0.0060	0.070
	0.00480	0.38	0.105	0.000666	0.01179	0.0060	0.070
	0.00170	0.38	0.088	0.000284	0.01179	0.0060	0.070
	0.00480	0.38	0.088	0.001340	0.01179	0.0060	0.070
	0.00480	0.38	0.298	0.000020	0.03769	0.0060	0.070
	0.00480	0.38	0.140	0.000366	0.03769	0.0060	0.070

	0.00170	0.38	0.140	0.000047	0.03769	0.0060	0.070
	0.00170	0.38	0.105	0.000232	0.03769	0.0060	0.070
**Nezu and Sanjou [26]**	0.00720	0.40	0.150	0.000777	0.18478	0.0080	0.050
	0.00720	0.40	0.150	0.000652	0.09239	0.0080	0.050
	0.00720	0.40	0.150	0.000544	0.04760	0.0080	0.050
	0.00250	0.40	0.063	0.001553	0.04760	0.0080	0.050
	0.00300	0.40	0.075	0.001165	0.04760	0.0080	0.050
	0.00400	0.40	0.100	0.000653	0.04760	0.0080	0.050
	0.00500	0.40	0.125	0.000460	0.04760	0.0080	0.050
	0.00600	0.40	0.150	0.000364	0.04760	0.0080	0.050
	0.00800	0.40	0.200	0.000196	0.04760	0.0080	0.050
**Poggi et al. [46]**	0.16200	0.90	0.600	0.000040	0.00084	0.0040	0.120
	0.16200	0.90	0.600	0.000070	0.00168	0.0040	0.120
	0.16200	0.90	0.600	0.000110	0.00337	0.0040	0.120
	0.16200	0.90	0.600	0.000180	0.00674	0.0040	0.120
	0.16200	0.90	0.600	0.000320	0.01347	0.0040	0.120
**Shimizu et al. [62]**	0.00207	0.50	0.064	0.000660	0.00785	0.0010	0.041
	0.00349	0.50	0.073	0.001080	0.00785	0.0010	0.041
	0.00479	0.50	0.088	0.000900	0.00785	0.0010	0.041
	0.00606	0.50	0.095	0.001000	0.00785	0.0010	0.041
	0.00774	0.50	0.105	0.000990	0.00785	0.0010	0.041
	0.00354	0.50	0.063	0.001640	0.00785	0.0010	0.041
	0.00518	0.50	0.075	0.002130	0.00785	0.0010	0.041
	0.00684	0.50	0.084	0.002010	0.00785	0.0010	0.041
	0.00856	0.50	0.094	0.001830	0.00785	0.0010	0.041
	0.01055	0.50	0.106	0.001760	0.00785	0.0010	0.041
	0.00478	0.50	0.066	0.002330	0.00785	0.0010	0.041
	0.00631	0.50	0.074	0.002630	0.00785	0.0010	0.041
	0.00851	0.50	0.085	0.003040	0.00785	0.0010	0.041
	0.01051	0.50	0.095	0.002560	0.00785	0.0010	0.041
	0.01415	0.50	0.103	0.003200	0.00785	0.0010	0.041
	0.00613	0.50	0.066	0.004550	0.00785	0.0010	0.041
	0.00754	0.50	0.074	0.004550	0.00785	0.0010	0.041
	0.00980	0.50	0.084	0.004350	0.00785	0.0010	0.041
	0.01294	0.50	0.096	0.004350	0.00785	0.0010	0.041
	0.01602	0.50	0.105	0.004760	0.00785	0.0010	0.041
	0.00504	0.40	0.095	0.001000	0.00442	0.0015	0.046
	0.00351	0.40	0.075	0.001000	0.00442	0.0015	0.046
	0.00733	0.40	0.094	0.003000	0.00442	0.0015	0.046
	0.00527	0.40	0.074	0.003000	0.00442	0.0015	0.046
	0.00216	0.40	0.050	0.003000	0.00442	0.0015	0.046
	0.00281	0.40	0.057	0.003000	0.00442	0.0015	0.046
	0.01183	0.40	0.090	0.007000	0.00442	0.0015	0.046

	0.00776	0.40	0.073	0.007000	0.00442	0.0015	0.046
**Stone and Shen [63]**	0.00570	0.45	0.151	0.002320	0.06106	0.0130	0.124
	0.00320	0.45	0.155	0.000910	0.06106	0.0130	0.124
	0.00480	0.45	0.155	0.001590	0.06106	0.0130	0.124
	0.00820	0.45	0.155	0.004060	0.06106	0.0130	0.124
	0.01100	0.45	0.155	0.007610	0.06106	0.0130	0.124
	0.01700	0.45	0.155	0.017000	0.06106	0.0130	0.124
	0.02600	0.45	0.155	0.032000	0.06106	0.0130	0.124
	0.00240	0.45	0.155	0.000550	0.06106	0.0130	0.124
	0.00270	0.45	0.153	0.000590	0.06106	0.0130	0.124
	0.00430	0.45	0.155	0.001440	0.06106	0.0130	0.124
	0.00710	0.45	0.155	0.003340	0.06106	0.0130	0.124
	0.02900	0.45	0.155	0.044000	0.06106	0.0130	0.124
	0.00450	0.45	0.206	0.000450	0.06106	0.0130	0.124
	0.00600	0.45	0.207	0.000630	0.06106	0.0130	0.124
	0.00870	0.45	0.205	0.000940	0.06106	0.0130	0.124
	0.01200	0.45	0.205	0.001980	0.06106	0.0130	0.124
	0.01800	0.45	0.206	0.004450	0.06106	0.0130	0.124
	0.02900	0.45	0.207	0.012000	0.06106	0.0130	0.124
	0.02300	0.45	0.207	0.007420	0.06106	0.0130	0.124
	0.00690	0.45	0.207	0.000810	0.06106	0.0130	0.124
	0.00500	0.45	0.206	0.000590	0.06106	0.0130	0.124
	0.00660	0.45	0.209	0.000540	0.06106	0.0130	0.124
	0.00800	0.45	0.206	0.000900	0.06106	0.0130	0.124
	0.00920	0.45	0.207	0.001170	0.06106	0.0130	0.124
	0.01100	0.45	0.212	0.001340	0.06106	0.0130	0.124
	0.01000	0.45	0.311	0.000360	0.06106	0.0130	0.124
	0.01100	0.45	0.308	0.000540	0.06106	0.0130	0.124
	0.01600	0.45	0.308	0.000760	0.06106	0.0130	0.124
	0.02100	0.45	0.311	0.000930	0.06106	0.0130	0.124
	0.01300	0.45	0.314	0.000400	0.06106	0.0130	0.124
	0.02800	0.45	0.308	0.001880	0.06106	0.0130	0.124
	0.01300	0.45	0.308	0.000350	0.06106	0.0130	0.124
	0.01100	0.45	0.308	0.000470	0.06106	0.0130	0.124
	0.01500	0.45	0.311	0.000540	0.06106	0.0130	0.124
	0.00380	0.45	0.155	0.000350	0.02203	0.0130	0.124
	0.00490	0.45	0.155	0.000580	0.02203	0.0130	0.124
	0.00710	0.45	0.155	0.001030	0.02203	0.0130	0.124
	0.00890	0.45	0.155	0.001700	0.02203	0.0130	0.124
	0.01100	0.45	0.155	0.002750	0.02203	0.0130	0.124
	0.01700	0.45	0.155	0.005230	0.02203	0.0130	0.124
	0.02800	0.45	0.155	0.014000	0.02203	0.0130	0.124
	0.01800	0.45	0.155	0.005680	0.02203	0.0130	0.124

	0.02100	0.45	0.155	0.008380	0.02203	0.0130	0.124
	0.02300	0.45	0.155	0.010000	0.02203	0.0130	0.124
	0.01500	0.45	0.155	0.004520	0.02203	0.0130	0.124
	0.00660	0.45	0.155	0.000980	0.02203	0.0130	0.124
	0.00890	0.45	0.155	0.002070	0.02203	0.0130	0.124
	0.00710	0.45	0.155	0.001180	0.02203	0.0130	0.124
	0.01300	0.45	0.155	0.003140	0.02203	0.0130	0.124
	0.01900	0.45	0.155	0.006790	0.02203	0.0130	0.124
	0.02300	0.45	0.155	0.009520	0.02203	0.0130	0.124
	0.00460	0.45	0.207	0.000230	0.02203	0.0130	0.124
	0.00590	0.45	0.207	0.000270	0.02203	0.0130	0.124
	0.00690	0.45	0.207	0.000360	0.02203	0.0130	0.124
	0.00810	0.45	0.207	0.000630	0.02203	0.0130	0.124
	0.00940	0.45	0.207	0.000530	0.02203	0.0130	0.124
	0.01100	0.45	0.207	0.000710	0.02203	0.0130	0.124
	0.01700	0.45	0.207	0.001530	0.02203	0.0130	0.124
	0.02900	0.45	0.207	0.004280	0.02203	0.0130	0.124
	0.02500	0.45	0.207	0.003820	0.02203	0.0130	0.124
	0.02100	0.45	0.207	0.002340	0.02203	0.0130	0.124
	0.00680	0.45	0.207	0.000350	0.02203	0.0130	0.124
	0.01500	0.45	0.207	0.001230	0.02203	0.0130	0.124
	0.00770	0.45	0.207	0.000540	0.02203	0.0130	0.124
	0.01300	0.45	0.207	0.000890	0.02203	0.0130	0.124
	0.01900	0.45	0.207	0.001950	0.02203	0.0130	0.124
	0.02400	0.45	0.207	0.003630	0.02203	0.0130	0.124
	0.01100	0.45	0.308	0.000450	0.02203	0.0130	0.124
	0.00950	0.45	0.308	0.000090	0.02203	0.0130	0.124
	0.01300	0.45	0.308	0.000360	0.02203	0.0130	0.124
	0.01500	0.45	0.308	0.000450	0.02203	0.0130	0.124
	0.01700	0.45	0.308	0.000540	0.02203	0.0130	0.124
	0.02700	0.45	0.308	0.000790	0.02203	0.0130	0.124
	0.02900	0.45	0.308	0.001470	0.02203	0.0130	0.124
	0.01200	0.45	0.308	0.000220	0.02203	0.0130	0.124
	0.01900	0.45	0.308	0.000400	0.02203	0.0130	0.124
	0.02400	0.45	0.308	0.000660	0.02203	0.0130	0.124
	0.00660	0.45	0.155	0.000980	0.00550	0.0032	0.124
	0.00760	0.45	0.155	0.001160	0.00550	0.0032	0.124
	0.01000	0.45	0.155	0.001870	0.00550	0.0032	0.124
	0.01400	0.45	0.155	0.004570	0.00550	0.0032	0.124
	0.01900	0.45	0.155	0.004430	0.00550	0.0032	0.124
	0.02800	0.45	0.155	0.011000	0.00550	0.0032	0.124
	0.00550	0.45	0.155	0.000540	0.00550	0.0032	0.124
	0.01100	0.45	0.155	0.002050	0.00550	0.0032	0.124

	0.00390	0.45	0.154	0.000260	0.00550	0.0032	0.124
	0.02200	0.45	0.155	0.006760	0.00550	0.0032	0.124
	0.00610	0.45	0.155	0.000760	0.00550	0.0032	0.124
	0.00750	0.45	0.155	0.000840	0.00550	0.0032	0.124
	0.01000	0.45	0.155	0.001730	0.00550	0.0032	0.124
	0.01100	0.45	0.155	0.001870	0.00550	0.0032	0.124
	0.02200	0.45	0.155	0.006940	0.00550	0.0032	0.124
	0.00370	0.45	0.207	0.000270	0.00550	0.0032	0.124
	0.00670	0.45	0.208	0.000170	0.00550	0.0032	0.124
	0.01100	0.45	0.209	0.000530	0.00550	0.0032	0.124
	0.01400	0.45	0.206	0.001060	0.00550	0.0032	0.124
	0.02800	0.45	0.206	0.003720	0.00550	0.0032	0.124
	0.02200	0.45	0.207	0.002310	0.00550	0.0032	0.124
	0.02400	0.45	0.206	0.002730	0.00550	0.0032	0.124
	0.01900	0.45	0.208	0.001620	0.00550	0.0032	0.124
	0.00890	0.45	0.205	0.000260	0.00550	0.0032	0.124
	0.02700	0.45	0.205	0.003650	0.00550	0.0032	0.124
	0.05400	0.45	0.205	0.015000	0.00550	0.0032	0.124
	0.01600	0.45	0.308	0.000400	0.00550	0.0032	0.124
	0.02200	0.45	0.308	0.000570	0.00550	0.0032	0.124
	0.02700	0.45	0.308	0.000880	0.00550	0.0032	0.124
	0.04200	0.45	0.308	0.002030	0.00550	0.0032	0.124
	0.06500	0.45	0.308	0.005220	0.00550	0.0032	0.124
	0.02400	0.45	0.311	0.000530	0.00550	0.0032	0.124
	0.00980	0.45	0.308	0.000090	0.00550	0.0032	0.124
	0.01700	0.45	0.308	0.000170	0.00550	0.0032	0.124
	0.02700	0.45	0.308	0.000880	0.00550	0.0032	0.124
	0.05400	0.45	0.311	0.003080	0.00550	0.0032	0.124
	0.01100	0.45	0.155	0.001080	0.00551	0.0064	0.124
	0.02700	0.45	0.155	0.007030	0.00551	0.0064	0.124
	0.02000	0.45	0.155	0.004130	0.00551	0.0064	0.124
	0.01700	0.45	0.155	0.002550	0.00551	0.0064	0.124
	0.00950	0.45	0.155	0.000830	0.00551	0.0064	0.124
	0.00870	0.45	0.205	0.000260	0.00551	0.0064	0.124
	0.01400	0.45	0.205	0.000610	0.00551	0.0064	0.124
	0.02000	0.45	0.205	0.001270	0.00551	0.0064	0.124
	0.02800	0.45	0.205	0.002390	0.00551	0.0064	0.124
	0.03900	0.45	0.205	0.004940	0.00551	0.0064	0.124
	0.05800	0.45	0.205	0.009510	0.00551	0.0064	0.124
	0.02100	0.45	0.310	0.000340	0.00551	0.0064	0.124
	0.02800	0.45	0.310	0.000450	0.00551	0.0064	0.124
	0.04000	0.45	0.310	0.001170	0.00551	0.0064	0.124
	0.05700	0.45	0.310	0.002570	0.00551	0.0064	0.124

**Yan [64]**	0.01440	0.42	0.120	0.012800	0.05655	0.0060	0.060
	0.02320	0.42	0.180	0.004800	0.05655	0.0060	0.060
	0.03100	0.42	0.240	0.002200	0.05655	0.0060	0.060
	0.03780	0.42	0.300	0.001200	0.05655	0.0060	0.060
	0.01460	0.42	0.120	0.007200	0.02827	0.0060	0.060
	0.02270	0.42	0.180	0.003100	0.02827	0.0060	0.060
	0.03020	0.42	0.240	0.001500	0.02827	0.0060	0.060
	0.03680	0.42	0.300	0.001100	0.02827	0.0060	0.060
	0.01510	0.42	0.120	0.003700	0.01414	0.0060	0.060
	0.02270	0.42	0.180	0.002600	0.01414	0.0060	0.060
	0.03020	0.42	0.240	0.001100	0.01414	0.0060	0.060
	0.03680	0.42	0.300	0.000650	0.01414	0.0060	0.060
**Yang and Choi [65]**	0.00750	0.45	0.075	0.001410	0.00440	0.0020	0.035
	0.01050	0.45	0.075	0.002690	0.00440	0.0020	0.035

Table S3. Experimental data of flow through submerged flexible vegetation

Author	*Q* (m^3/s)	*B* (m)	h_w (m)	S_o	ϕ	*D* (m)	$h_{v\text{-}bend}$ (m)
Dunn [58]	0.1790	0.91	0.3671	0.00360	0.00545	0.00635	0.1520
	0.1800	0.91	0.2318	0.01010	0.00545	0.00635	0.1150
	0.0930	0.91	0.2571	0.00360	0.00545	0.00635	0.1320
	0.1790	0.91	0.2301	0.00360	0.00136	0.00635	0.0970
	0.0780	0.91	0.2785	0.00360	0.01229	0.00635	0.1610
	0.1790	0.91	0.2835	0.01010	0.01229	0.00635	0.1210
Yang and Choi [65]	0.0075	0.45	0.0550	0.00361	0.00440	0.00200	0.0226
	0.0075	0.45	0.0750	0.00151	0.00440	0.00200	0.0275
	0.0105	0.45	0.0750	0.00266	0.00440	0.00200	0.0253
	0.0075	0.45	0.1100	0.00070	0.00440	0.00200	0.0339
	0.0105	0.45	0.1100	0.00079	0.00440	0.00200	0.0309
Kubrak et al. [66]	0.0433	0.58	0.2661	0.00870	0.00535	0.00083	0.1630
	0.0384	0.58	0.2576	0.00870	0.00535	0.00083	0.1630
	0.0333	0.58	0.2475	0.00870	0.00535	0.00083	0.1640
	0.0274	0.58	0.2275	0.00870	0.00535	0.00083	0.1640
	0.0422	0.58	0.2236	0.01740	0.00535	0.00083	0.1610
	0.0385	0.58	0.2184	0.01740	0.00535	0.00083	0.1620
	0.0333	0.58	0.2068	0.01740	0.00535	0.00083	0.1610
	0.0274	0.58	0.1951	0.01740	0.00535	0.00083	0.1620
	0.0525	0.58	0.2386	0.00870	0.00134	0.00083	0.1530
	0.0425	0.58	0.2136	0.00870	0.00134	0.00083	0.1540
	0.0332	0.58	0.1935	0.00870	0.00134	0.00083	0.1550
	0.0751	0.58	0.2131	0.01740	0.00134	0.00083	0.1320
	0.0650	0.58	0.1925	0.01740	0.00134	0.00083	0.1310
	0.0547	0.58	0.1799	0.01740	0.00134	0.00083	0.1330
	0.0605	0.58	0.2386	0.00870	0.00134	0.00083	0.1510
	0.0504	0.58	0.2234	0.00870	0.00134	0.00083	0.1520
	0.0408	0.58	0.2005	0.00870	0.00134	0.00083	0.1530
	0.0693	0.58	0.1962	0.01740	0.00134	0.00083	0.1320
	0.0555	0.58	0.1876	0.01740	0.00134	0.00083	0.1390
	0.0609	0.58	0.2421	0.00870	0.00134	0.00083	0.1510
	0.0500	0.58	0.2246	0.00870	0.00134	0.00083	0.1530
	0.0408	0.58	0.2053	0.00870	0.00134	0.00083	0.1560
	0.0693	0.58	0.2077	0.01740	0.00134	0.00083	0.1380
	0.0466	0.58	0.1932	0.01740	0.00134	0.00083	0.1420
	0.0553	0.58	0.1806	0.01740	0.00134	0.00083	0.1430
Okamoto and Nezu [67]	0.0210	0.40	0.1500	0.00241	0.04780	0.00800	0.0300
	0.0180	0.40	0.1500	0.00221	0.04780	0.00800	0.0340
	0.0150	0.40	0.1500	0.00200	0.04780	0.00800	0.0360
	0.0120	0.40	0.1500	0.00165	0.04780	0.00800	0.0400

	0.0102	0.40	0.1500	0.00141	0.04780	0.00800	0.0420
	0.0090	0.40	0.1500	0.00113	0.04780	0.00800	0.0440
	0.0072	0.40	0.1500	0.00078	0.04780	0.00800	0.0460
	0.0060	0.40	0.1500	0.00056	0.04780	0.00800	0.0490
	0.0294	0.40	0.2100	0.00149	0.04780	0.00800	0.0400
	0.0252	0.40	0.2100	0.00137	0.04780	0.00800	0.0450
	0.0210	0.40	0.2100	0.00129	0.04780	0.00800	0.0510
	0.0168	0.40	0.2100	0.00106	0.04780	0.00800	0.0560
	0.0143	0.40	0.2100	0.00086	0.04780	0.00800	0.0580
	0.0126	0.40	0.2100	0.00074	0.04780	0.00800	0.0600
	0.0101	0.40	0.2100	0.00051	0.04780	0.00800	0.0630
	0.0084	0.40	0.2100	0.00038	0.04780	0.00800	0.0680
	0.0270	0.40	0.2700	0.00062	0.04780	0.00800	0.0540
	0.0216	0.40	0.2700	0.00054	0.04780	0.00800	0.0600
	0.0184	0.40	0.2700	0.00049	0.04780	0.00800	0.0650
	0.0162	0.40	0.2700	0.00044	0.04780	0.00800	0.0710
	0.0130	0.40	0.2700	0.00033	0.04780	0.00800	0.0750
	0.0108	0.40	0.2700	0.00024	0.04780	0.00800	0.0780
	0.0315	0.40	0.3150	0.00041	0.04780	0.00800	0.0640
	0.0252	0.40	0.3150	0.00039	0.04780	0.00800	0.0680
	0.0214	0.40	0.3150	0.00038	0.04780	0.00800	0.0760
	0.0189	0.40	0.3150	0.00033	0.04780	0.00800	0.0810
	0.0151	0.40	0.3150	0.00025	0.04780	0.00800	0.0840
	0.0126	0.40	0.3150	0.00019	0.04780	0.00800	0.0960
**Järvelä [68]**	0.0400	1.10	0.3060	0.00150	0.07389	0.00280	0.2050
	0.1000	1.10	0.3084	0.00360	0.07389	0.00280	0.1550
	0.0400	1.10	0.4065	0.00050	0.07389	0.00280	0.2300
	0.1000	1.10	0.4041	0.00130	0.07389	0.00280	0.1900
	0.1430	1.10	0.4070	0.00200	0.07389	0.00280	0.1600
	0.0400	1.10	0.5044	0.00020	0.07389	0.00280	0.2450
	0.1000	1.10	0.4950	0.00060	0.07389	0.00280	0.2200
	0.1000	1.10	0.7065	0.00020	0.07389	0.00280	0.2600
	0.1430	1.10	0.7037	0.00030	0.07389	0.00280	0.2150
**Carollo et al. [69]**	0.0376	0.60	0.1190	0.01000	0.02435	0.00100	0.0480
	0.0301	0.60	0.1350	0.01000	0.03456	0.00100	0.0800
	0.0301	0.60	0.1460	0.00200	0.03456	0.00100	0.0800
	0.0269	0.60	0.1400	0.00200	0.03456	0.00100	0.0820
	0.0269	0.60	0.1250	0.01000	0.03456	0.00100	0.0770
	0.0776	0.60	0.1780	0.00200	0.03456	0.00100	0.0700
	0.0776	0.60	0.1680	0.01000	0.03456	0.00100	0.0660
	0.1059	0.60	0.1990	0.00200	0.03456	0.00100	0.0630
	0.1059	0.60	0.1830	0.01000	0.03456	0.00100	0.0590
	0.0269	0.60	0.1280	0.00200	0.02199	0.00100	0.0700

	0.0776	0.60	0.1900	0.00200	0.02199	0.00100	0.0540
	0.1059	0.60	0.2170	0.00200	0.02199	0.00100	0.0490
	0.1350	0.60	0.2450	0.00200	0.02199	0.00100	0.0470
	0.1708	0.60	0.2720	0.00200	0.02199	0.00100	0.0450
	0.1887	0.60	0.2770	0.00200	0.02647	0.00100	0.0380
	0.1892	0.60	0.2720	0.00200	0.02647	0.00100	0.0310
**Ciraolo and Ferreri [11]**	0.0574	0.77	0.1500	0.00850	0.02036	0.00500	0.0680
	0.0681	0.77	0.1610	0.00811	0.02036	0.00500	0.0630
	0.0750	0.77	0.2490	0.00198	0.02036	0.00500	0.1050
	0.0951	0.77	0.2430	0.00291	0.02036	0.00500	0.0950
	0.1099	0.77	0.2450	0.00397	0.02036	0.00500	0.1000
	0.0270	0.77	0.3600	0.00009	0.02036	0.00500	0.2350
	0.0550	0.77	0.3670	0.00032	0.02036	0.00500	0.1600
	0.0840	0.77	0.3650	0.00061	0.02036	0.00500	0.1400
	0.1161	0.77	0.3480	0.00103	0.02036	0.00500	0.1180
	0.1346	0.77	0.3500	0.00138	0.02036	0.00500	0.1150
	0.1574	0.77	0.3480	0.00174	0.02036	0.00500	0.1050
	0.0349	0.77	0.4660	0.00008	0.02036	0.00500	0.2900
	0.0689	0.77	0.4700	0.00019	0.02036	0.00500	0.1900
	0.1081	0.77	0.4780	0.00045	0.02036	0.00500	0.2350
	0.1456	0.77	0.4740	0.00055	0.02036	0.00500	0.1150
	0.1770	0.77	0.4650	0.00081	0.02036	0.00500	0.1450
**Kouwen et al. [70]**	0.0027	0.61	0.1506	0.00050	0.09817	0.00500	0.1000
	0.0169	0.61	0.2527	0.00100	0.09817	0.00500	0.1000
	0.0856	0.61	0.3819	0.00300	0.09817	0.00500	0.0850
	0.0091	0.61	0.1519	0.00500	0.09817	0.00500	0.1000
	0.0132	0.61	0.1509	0.01000	0.09817	0.00500	0.1000
	0.0827	0.61	0.2422	0.00940	0.09817	0.00500	0.0500
	0.0437	0.61	0.3503	0.00100	0.09817	0.00500	0.1000
	0.0408	0.61	0.2500	0.00490	0.09817	0.00500	0.1000
	0.0381	0.61	0.4000	0.00050	0.09817	0.00500	0.1000
	0.0194	0.61	0.3000	0.00050	0.09817	0.00500	0.1000
	0.0067	0.61	0.2002	0.00050	0.09817	0.00500	0.1000
	0.0496	0.61	0.3000	0.00300	0.09817	0.00500	0.0950
	0.0097	0.61	0.2001	0.00100	0.09817	0.00500	0.1000
	0.0479	0.61	0.1990	0.01000	0.09817	0.00500	0.0600
	0.0284	0.61	0.3498	0.00050	0.09817	0.00500	0.1000
	0.0731	0.61	0.2998	0.00500	0.09817	0.00500	0.0750
	0.0288	0.61	0.3000	0.00100	0.09817	0.00500	0.1000
	0.0165	0.61	0.2000	0.00300	0.09817	0.00500	0.1000
	0.0225	0.61	0.2000	0.00500	0.09817	0.00500	0.1000
	0.1139	0.61	0.3486	0.00500	0.09817	0.00500	0.0600
	0.0558	0.61	0.3986	0.00100	0.09817	0.00500	0.0900

	0.0127	0.61	0.2527	0.00050	0.09817	0.00500	0.1000
	0.0754	0.61	0.3508	0.00300	0.09817	0.00500	0.0900
	0.0310	0.61	0.2594	0.00300	0.09817	0.00500	0.1000
	0.1422	0.61	0.3830	0.00490	0.09817	0.00500	0.0550
	0.0038	0.61	0.1491	0.00100	0.09817	0.00500	0.1000
